# The functional consequences of age-related changes in microRNA expression in skeletal muscle

**DOI:** 10.1007/s10522-016-9638-8

**Published:** 2016-02-27

**Authors:** Ana Soriano-Arroquia, Louise House, Luke Tregilgas, Elizabeth Canty-Laird, Katarzyna Goljanek-Whysall

**Affiliations:** MRC-Arthritis Research UK Centre for Integrated research into Musculoskeletal Ageing (CIMA), Department of Musculoskeletal Biology, Institute of Ageing and Chronic Disease, University of Liverpool, Apex Building, West Derby Road, Liverpool, L8 7TX UK

**Keywords:** microRNA, Sarcopenia, *Sirt1*, Muscle, Ageing

## Abstract

A common characteristic of ageing is disrupted homeostasis between growth and atrophy of skeletal muscle resulting in loss of muscle mass and function, which is associated with sarcopenia. Sarcopenia is related to impaired balance, increased falls and decline in quality of life of older people. Ageing-related transcriptome and proteome changes in skeletal muscle have been characterised, however the molecular mechanisms underlying sarcopenia are still not fully understood. microRNAs are novel regulators of gene expression known to modulate skeletal muscle development and homeostasis. Expression of numerous microRNAs is disrupted in skeletal muscle with age however, the functional consequences of this are not yet understood. Given that a single microRNA can simultaneously affect multiple signalling pathways, microRNAs are potent modulators of pathophysiological changes occurring during ageing. Here we use microRNA and transcript expression profiling together with microRNA functional assays to show that disrupted microRNA:target interactions play an important role in maintaining muscle homeostasis. We identified miR-181a as a regulator of the sirtuin1 (*Sirt1*) gene expression in skeletal muscle and show that the expression of miR-181a and its target gene is disrupted in skeletal muscle from old mice. Moreover, we show that miR-181a:*Sirt1* interactions regulate myotube size. Our results demonstrate that disrupted microRNA:target interactions are likely related to the pathophysiological changes occurring in skeletal muscle during ageing.

**Electronic supplementary material:**

The online version of this article (doi:10.1007/s10522-016-9638-8) contains supplementary material, which is available to authorized users.

## Introduction

Age-related loss of skeletal muscle mass and function, associated with sarcopenia, results in frailty, decline in strength and decrease in quality of life of older people. The mechanisms of age-related defective muscle homeostasis related to hypertrophy/atrophy are multifactorial and depend on a number of changes including mitochondrial production of reactive oxygen species (Jackson and McArdle 2011), changes in the muscle niche (Carlson and Conboy 2007), or alterations in circulating factors (Carlson et al. 2009). Moreover, skeletal muscles of adult and old animals display substantially different gene (Welle et al. 2003; Sifakis et al. 2013) and protein (McDonagh et al. 2014) expression profiles.

microRNAs (miRNAs) are small, non-coding RNAs that regulate gene expression at the post-transcriptional level. miRNAs are predicted to regulate two-thirds of the human genome, suggesting that miRNAs modulate many physiologically relevant processes (Friedman et al. 2009). Mature miRNAs are generated from primary-miRNA (pri-miRNA) precursors which are cleaved by the enzyme Drosha forming the pre-miRNA transcript. The pre-miRNA is transported into the cytoplasm, where the enzyme Dicer generates a 19–24 base pair miRNA duplex (Bartel 2004). The mature miRNA strand is incorporated into the RISC complex (RNA Induced Silencing Complex). The non-incorporated strand is often degraded however it may also be incorporated into the RISC. miRNAs guide the RISC to partially complementary sequences, usually contained within the 3′ UTR of target mRNA transcripts, resulting in target mRNA degradation and/or inhibition of translation. Most mammalian miRNAs have only partially complementary sequences to their target mRNAs (Bartel 2004) resulting in challenging bioinformatic prediction of the target genes.

microRNAs play significant roles in myogenic processes during embryonic development (Goljanek-Whysall et al. 2011, 2014) and in adults by regulating satellite cell function, muscle hypertrophy and myofibre type (McCarthy and Esser 2007; Crist et al. 2009; Van Rooij et al. 2009b; Goljanek-Whysall et al. 2012; Brown and Goljanek-Whysall 2015; Soriano-Arroquia et al. 2016). Moreover, the role of miRNAs in muscle during ageing has been demonstrated using satellite cell specific Dicer-deficient mice (Cheung et al. 2012), characterised by mild muscle atrophy and an impaired ability to regenerate muscle fibres following muscle injury. The differential expression of many muscle-enriched as well as non-muscle specific miRNAs in skeletal muscle during ageing in multiple species, including mice (Kim et al. 2014a), rats (Hu et al. 2014a), rhesus monkeys (Mercken et al. 2013) and humans (Drummond et al. 2011a; Rivas et al. 2014b; Zacharewicz et al. 2014) has been demonstrated. Moreover, differential expression of miRNAs in skeletal muscle of adult and old humans in response to an acute bout of resistance exercise has been shown (Rivas et al. 2014a; Zacharewicz et al. 2014). Soares et al. (2014) recently demonstrated that different catabolic conditions in muscle are associated with a unique and dynamic miRNA signature (Soares et al. 2014).

In this study, we aimed to establish the potential functional consequences of age-related dysregulation of microRNA:target interactions in skeletal muscle during ageing. We characterised the global changes in microRNA and mRNA expression profiles in skeletal muscle of adult and old mice using bioinformatics tools and modelling. Moreover, we identified a group of microRNAs, expression of which is affected by ageing in mice and humans and validated the ageing-associated changes in their expression in mouse skeletal muscle. We demonstrated that miR-181a is predicted to play a central role in ageing-related disrupted muscle homeostasis and validated differential expression of microRNA-181a and its target genes in skeletal muscle during ageing. Finally, we validated *Sirt1* as a miR-181a target and demonstrated that manipulation of miR-181a expression regulates myotube size in vitro. These data suggest that ageing-associated changes in miR-181a and expression of its target gene(s) may indeed be associated with the ageing-related disrupted balance between muscle hypertrophy and atrophy.

## Materials and methods

### Mice

The study was performed using male wild type C57Bl/6 mice obtained from Charles River (Margate). All mice were maintained under specific-pathogen free conditions, fed ad libitum a standard chow and maintained under barrier on a 12-h light–dark cycle. For tissue collection, mice were culled by cervical dislocation. The tissues were immediately excised, frozen and stored at −80 °C. Experiments were performed in accordance with UK Home Office guidelines under the UK Animals (Scientific Procedures) Act 1986 and received ethical approval from the University of Liverpool Animal Welfare and Ethical Review Body (AWERB). For microarray studies, n = 3 adult (6 month) and n = 3 old (24 month) mice were used; for qPCR validation, an independent cohort of n = 6–9 adult and n = 4–6 old mice was used.

### microRNA and mRNA expression profiling in skeletal muscle of adult and old mice

The tibialis anterior muscles (30–40 mg each) from adult and old mice were dissected and RNA was isolated using Trizol as described below. RNA integrity was assessed using a Bioanalyser instrument (Agilent Technologies) and samples with RIN > 7 were used. An Affymetrix FlashTag Labelling kit and GeneChip microRNA 3.0 arrays were used for global miRNA expression profiling. GeneChip Mouse Gene 2.0 ST arrays (Affymetrix) were used for global transcriptomic analysis. Data pre-processing, modelling and differential expression analyses were carried out using the Expression Console and Transcriptome Analysis Control (Affymetrix) in the R environment (http://cran.r-project.org) using packages from the Bioconductor project (http://www.bioconductor.org). The oligo package (Carvalho and Irizarry 2010) has been developed for use with Affymetrix Gene ST arrays and takes into account the lack of incorporation of mismatch probes into the chip architecture. Raw Data distributions were initially visualised and assessed for spatial artefacts, array clustering, technical variability between arrays and to identify outliers prior to carrying out background correction, normalisation and summarisation using Robust Multichip Averaging (RMA). There were no outlying samples identified in the dataset, therefore n = 3 persists through to the differential expression analysis and the full sample set is represented. Data was then filtered for low variance using the genefilter package. Modelling and statistical analysis of differentially expressed genes was carried out in the limma package (Smyth 2004; Smyth et al. 2005; Ritchie et al. 2006, 2015). Two-group comparisons were used to determine statistically significant, differentially expressed transcript clusters using an empirical Bayes moderated *t* test. The Benjamini-Hochberg method was used for false discovery rate correction of p-values. For changes in microRNA expression in human skeletal muscle, publically available datasets were used (GSE23527; (Drummond et al. 2011b; Rivas et al. 2014a).

### microRNA:target interaction predictions

Analysis of differentially expressed microRNAs and mRNAs and predicted microRNA target genes was completed using QIAGEN’s Ingenuity^®^ Pathway Analysis (IPA^®^) Product (Ingenuity^®^ Systems, http://www.ingenuity.com), specifically the Core Analysis function, the Path Designer feature and the microRNA Target Filter. A list of experimentally validated and predicted microRNA targets was produced based on Targetscan Human, TarBase, miRecords, Ingenuity^®^ Expert Findings and Ingenuity^®^ ExpertAssist Findings.

### Cell culture and transfections

C2C12 cells were cultured in Dulbecco’s modified medium (DMEM) supplemented with 10 % fetal bovine serum (FBS), 1 % l-glutamine and 1 % penicillin/streptomycin. To induce myogenic differentiation, 90 % confluent cells were cultured in DMEM supplemented with 2 % horse serum, 1 % l-glutamine and 1 % penicillin/streptomycin (Goljanek-Whysall et al. 2012).

Myotubes were transfected with either 100 nM miRNA-181, 100 nM antimiR-181 or 2.5 µg SIRT1 overexpression vector (Addgene, 1791) (Brunet et al. 2004) using Lipofectamine 2000™. Mock-transfected or antimiR-scrambled-transfected cells served as controls. Transfection efficiency was 40–50 %, depending on the molecule transfected as described previously (Goljanek-Whysall et al. 2012). To assess the changes in myotube diameter following the transfections, MF20 (myosin heavy chain) immunostaining was performed at 4 days post- transfection.

### Real-Time PCR and western blotting

RNA isolation and quantitative real time PCR were performed using standard methods as described previously (Goljanek-Whysall et al. 2014). cDNA synthesis (mRNA) was performed using 400–500 ng RNA and SuperScript II according to the manufacturer’s protocol. cDNA synthesis (microRNA) was performed using 100 ng RNA and miRscript RT kit II according to the manufacturer’s protocol. microRNA qPCR analysis was performed using miRScript SybrGreen Mastermix in a 20 μl reaction according to the manufacturer’s protocol. mRNA qPCR was performed using sso-Advanced SybrGreen Mastermix, using 1 μl of 10μM each primer in 20 μl reaction. All amplicons were designed to span an intron–exon junction (where mRNAs consisted of more than one exon) and were 200–300 nt long. Expression relative to *β*-*2 microglobulin* and/or *18S* (mRNA) or Rnu-6 and/or Snord-61 (microRNA) was calculated using the delta delta Ct method. The qPCR conditions were: 95 °C 10 s, 55 °C (miRNA) or 60 °C (mRNA) 30 s, 72 °C 30 s (40x). Protein lysis and Western blots were performed as described (Goljanek-Whysall et al. 2014). The primers and reagents used are listed in Tables S4 and S5, respectively.

### In vitro miRNA target validation

Predicted target genes of miR-181 were initially chosen based on the global profiling data. Next our own and published data were used to narrow down the list of potential candidate targets to the ones that may be relevant to muscle ageing. The 3′UTR region of *Sirt1* with a wild type (WT) or mutated miR-181 target site (mutant) were synthesised using GeneArt service (Invitrogen) and cloned into a GFP TOPO vector (Invitrogen). C2C12 myotubes cultured in 6-well plates were transfected using Lipofectamine 2000™ with WT or mutant constructs (1000 ng), with or without miRNA mimic (50 nM). Protein was extracted after 48 h and GFP expression analysed by Western blotting.

## Results

### Ageing affects microRNA:target interactions in skeletal muscle of mice

To model microRNA:target interactions in skeletal muscle during ageing, an unbiased microRNA and mRNA expression profiling was performed on the tibilais anterior (TA) muscle of adult and old mice using microarrays (Figure S1). The expression of 16 microRNAs was significantly downregulated and the expression of 14 microRNAs was significantly upregulated in muscle during ageing (Table 1). Among the differentially expressed microRNAs were miR-181a, miR-208-5p or miR-499, miR-130a, miR-26a and miR-30c with previously characterised functions in skeletal muscle.

**Table 1:** microRNAs differentially expressed in the tibialis anterior muscle of mice during ageing

microRNA downregulated during ageing	Fold Change (Old vs Adult)	p value	microRNA upregulated during ageing	Fold Change (Old vs Adult)	p value
miR-122	−19.64	0.039	miR-26a	1.49	0.0004
miR-382	−6.17	0.038	mir-499	1.48	0.001
miR-148a	−4.31	0.038	miR-5103	1.32	0.002
miR-344f-5p	−3.41	0.038	miR-34b-3p	3.15	0.002
miR-132	−2.63	0.003	miR-669 g	1.31	0.003
miR-379	−2.39	0.028	mir-669o	1.76	0.004
miR-301a	−2.26	0.044	miR-217*	1.12	0.005
miR-30c*	−2.06	0.00002	mir-5123	1.63	0.005
miR-127	−2.05	0.004	miR-1186	2.05	0.008
mir-181a	−1.52	0.001	miR-431*	2.51	0.009
miR-208a-5p	−1.5	0.00	mir-669 m	2.15	0.016
mir-3074-1	−1.36	0.005	miR-467b*	3.1	0.016
miR-471-3p	−1.32	0.002	miR-133a*	2.39	0.033
miR-3475	−1.29	0.004	miR-872*	2.23	0.049
miR-465c-3p	−1.19	0.0006			
mir-3063	−1.18	0.004			

microRNA expression was assessed in the TA muscle of adult and old mice; the microRNA identity and fold change are given. MicroRNAs previously noted to be differentially expressed in human skeletal muscle during ageing are highlighted in red; adult: 6 months old; old: 24 months old; n = 3

Given the potential of miRNAs to regulate a large number of cellular transcripts, transcript expression profiling was performed. The expression of only 12 transcripts was significantly downregulated and the expression of 19 mRNAs was significantly upregulated in the TA muscle of old mice compared with the muscle of adult mice (p < 0.05; Table 2). This low number of differentially expressed genes in muscle during ageing may be related to a limited number of biological replicates (n = 3). Among the genes differentially expressed during ageing were genes associated with metabolism and mitochondrial function: *Ucp3*, *Pdk4*; methylation: *Mettl21c*; DNA damage response: *Gadd45* *g*; retinoic acid signalling: *Rarres1*; as well as cellular senescence: *Cdkn1* (p21).

**Table 2:** Transcripts differentially expressed in the tibialis anterior muscle of mice during ageing

Gene Symbol (transcripts downregulated)	Fold change (Old vs Adult)	p value	Description
Krt10	−7.61	0.030813	keratin 10
Arrdc2	−4.87	0.034934	arrestin domain containing 2
Dsc1	−3.55	0.033618	desmocollin 1
Dsg1a	−3.51	0.042671	desmoglein 1 alpha
Krt77	−3.31	0.046219	keratin 77
Hrnr	−3.07	0.014021	hornerin
Gm24202	−3.05	0.04403	predicted gene, 24202
Pof1b	−2.93	0.039252	premature ovarian failure 1B
Mettl21c	−2.88	0.008667	methyltransferase like 21C
Ucp3	−2.75	0.003941	uncoupling protein 3 (mitochondrial, proton carrier)
Neto2	−2.61	0.046314	neuropilin (NRP) and tolloid (TLL)-like 2
Gm24128	−2.4	0.032397	predicted gene, 24128
Lce1 m	−2.39	0.014247	late cornified envelope 1 M
Slc25a30	−2.37	0.006328	solute carrier family 25, member 30
Gadd45g	−2.34	0.001227	growth arrest and DNA-damage-inducible 45 gamma
Nrep	−2.1	0.003032	neuronal regeneration related protein
Rarres1	−2.04	0.037197	retinoic acid receptor responder (tazarotene induced) 1
Zfp640	−2.02	0.03305	zinc finger protein 845-like; zinc finger protein 640
Pdk4	−2.01	0.002642	pyruvate dehydrogenase kinase, isoenzyme 4

Gene Symbol (transcripts upregulated)	Fold Change (Old vs Adult)	p-value	Description
Krt18	3.76	0.002333	keratin 18
Cpne2	3.02	0.002247	copine II
Igkv4-57-1	2.75	0.023636	immunoglobulin kappa variable 4-57-1
Nabp1	2.49	0.007488	nucleic acid binding protein 1
Cilp	2.48	0.002913	cartilage intermediate layer protein, nucleotide pyrophosphohydrolase
Tnfrsf23	2.41	0.005439	tumor necrosis factor receptor superfamily, member 23
Rrad	2.25	0.009309	Ras-related associated with diabetes
Cdkn1a	2.21	0.018982	cyclin-dependent kinase inhibitor 1A (P21)
Gm6821	2.19	0.009309	predicted gene 6821
Ankrd1	2.17	0.008222	ankyrin repeat domain 1 (cardiac muscle)
Gm26179	2.11	0.035369	predicted gene, 26179
Trim30a	2.1	0.006883	tripartite motif-containing 30A

Target name and description, fold change in expression and p value are given;* adult* 6 months old;* old* 24 months old; n = 3

To understand the biological relevance of the changes in microRNA expression during ageing, microRNA: predicted target interactions were modelled using miRSystem (http://mirsystem.cgm.ntu.edu.tw/index.php). The top pathway predicted to be regulated by microRNAs downregulated in muscle during ageing was the insulin signalling pathway with 137 microRNA putative targets being associated with this pathway (Table 3). Targets of microRNAs upregulated during ageing were associated with protein processing in endoplasmic reticulum, splicing and ubiquitin-mediated proteolysis (Table 3).

**Table 3:** Functional annotation of predicted targets of microRNAs differentially expressed in skeletal muscle of mice during ageing; term—functional annotation term associated with miRNA predicted target genes

TERM (Predicted targets of downregulated mirnas)	Total genes	p value
Insulin signaling pathway	137	4.05E−02
Carbohydrate digestion and absorption	39	4.06E−02
Starch and sucrose metabolism	44	4.45E−02
Type ii diabetes mellitus	49	4.95E−02
Amino sugar and nucleotide sugar metabolism	47	6.56E−02
Glycolysis gluconeogenesis	60	9.10E−02
Fructose and mannose metabolism	36	9.26E−02
Galactose metabolism	27	2.62E−01
TERM (Predicted targets of upregulated mirnas)		
Endoplasmic reticulum	167	3.01E−01
Rna transport	150	2.82E−01
Ubiquitin mediated proteolysis	138	1.59E−01
Spliceosome	126	3.25E−01
Erbb signaling pathway	87	6.63E−02

**Fig. 1:**
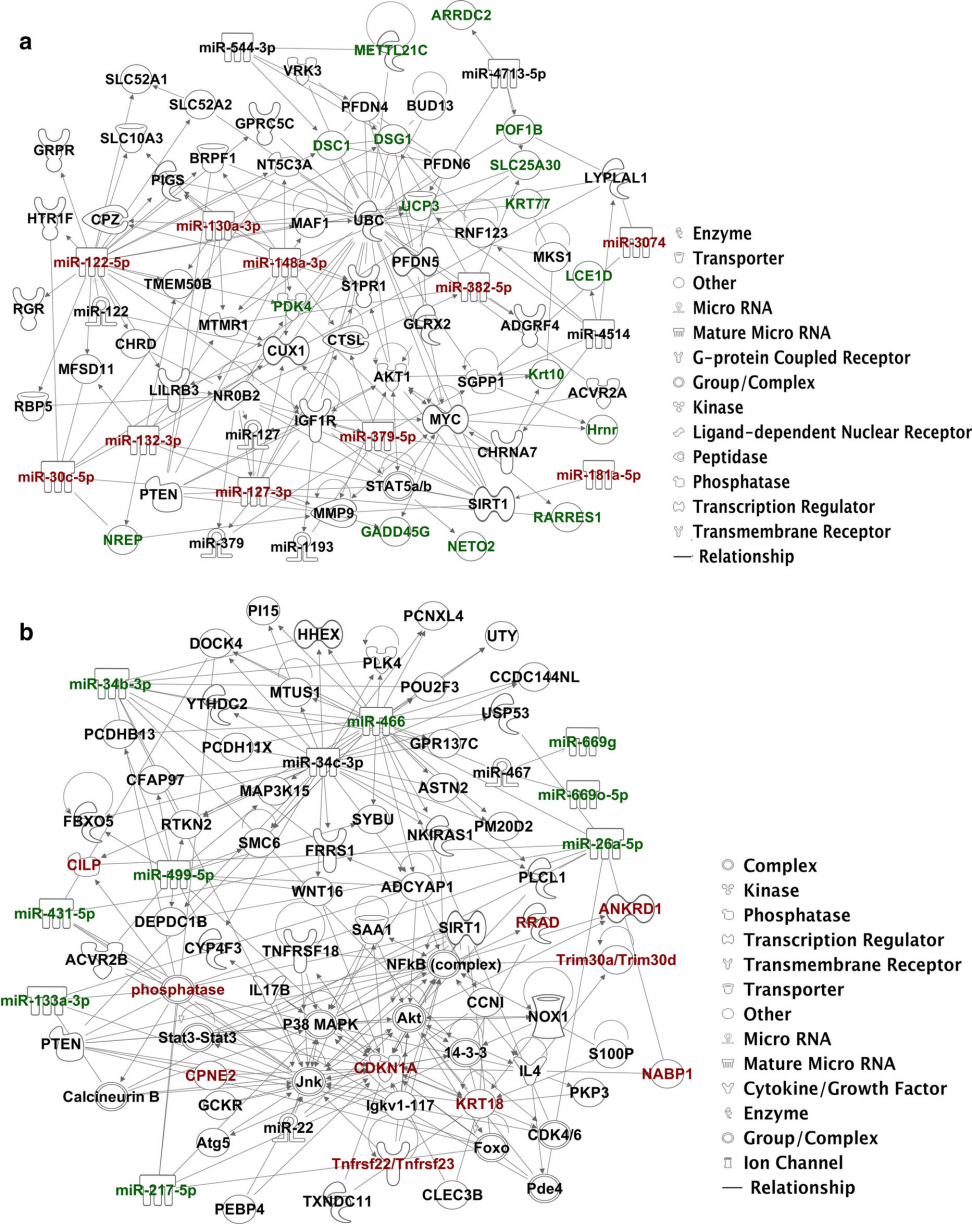
Disrupted microRNA:target interaction networks in skeletal muscle of mice during ageing. The network of interactions was constructed using IPA software. The inputs were microRNAs and their predicted targets and mRNAs differentially expressed in muscle during ageing. In red miRs/genes that were downregulated with age are shown; in green miRs/genes that were upregulated with age are shown. **a.** Networks of interactions associated with miRs downregulated in muscle during ageing. **b.** Networks of interactions associated with miRs upregulated in muscle during ageing

Analysis of microRNA:target interactions using Ingenuity pathways indicated that ubiquitin-mediated proteolysis, mitochondrial metabolism, insulin signalling and splicing were regulated by microRNAs downregulated in muscle during ageing, whereas cell senescence, TGFβ (transforming growth factor beta), NF-kB (nuclear factor κB) and MAPK (mitogen-activated protein kinase) signalling were mainly regulated by microRNAs upregulated in muscle during ageing (Fig. 1). Further pathway analysis using Ingenuity was performed to determine which miRNAs and their target genes form networks of connections. We observed that there were several genes and microRNAs forming nodes in the interaction network that comprised differentially expressed mRNAs and microRNAs and their predicted target genes (Fig. S2). Genes associated with ubiquitin-mediated proteolysis, cell cycle, NF-kB and insulin signalling, as well as miRs miR-132, miR-122, miR-499, miR-128 and miR-22 formed central nodes in the network of miRNA:target interactions disrupted during ageing (Fig. S2).

Overall, these data indicate that several miRNA:target interactions are deregulated in skeletal muscle during ageing. These changes are likely to contribute to ageing-related pathophysiological processes, such as loss of muscle mass and function through regulation of ubiquitin-mediated proteolysis, ER stress and NF-κb signalling, senescence associated with disrupted cell cycle or myofibre hypertrophy associated with insulin signalling.

### A set of microRNAs is deregulated during ageing in muscle of human and mice

**Fig. 2:**
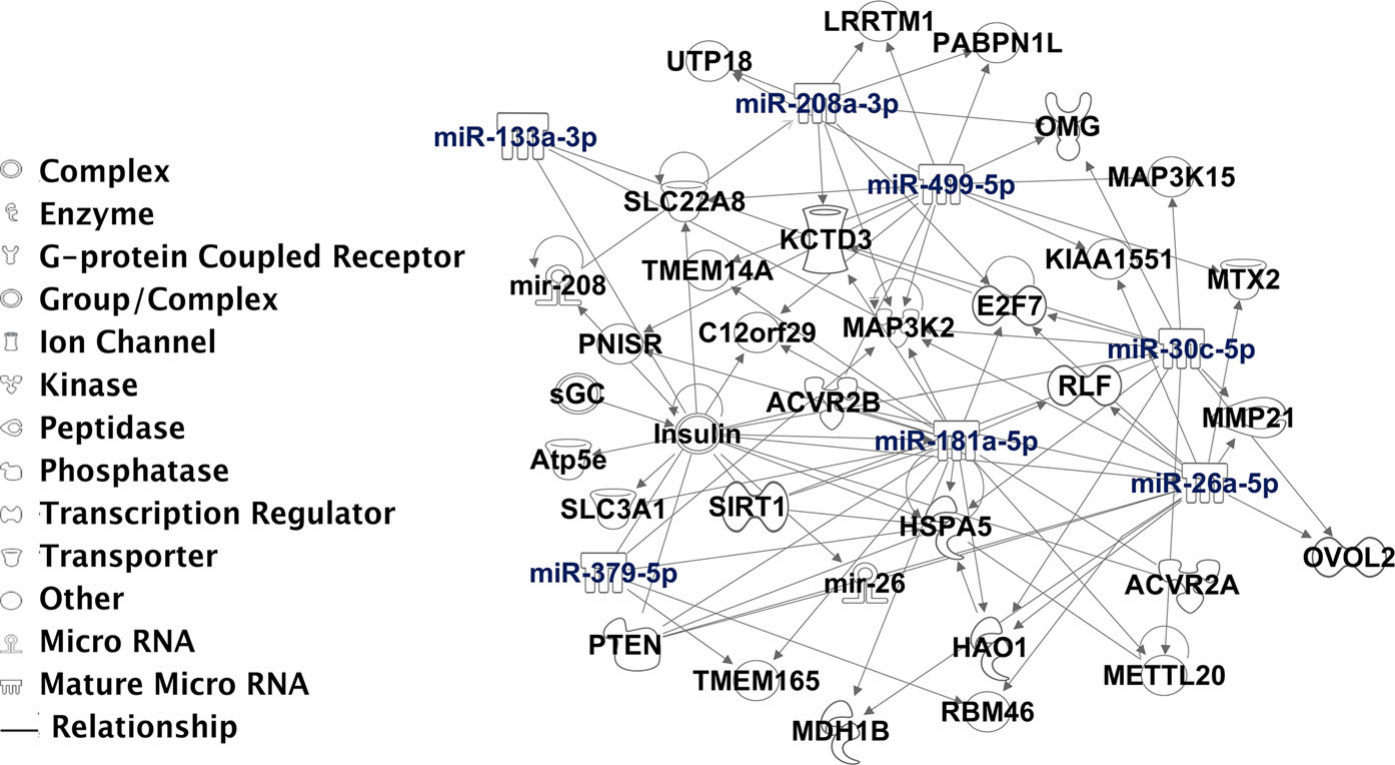
Disrupted microRNA:target interaction network in skeletal muscle of humans and mice during ageing. The network of interactions was constructed using IPA software. The input was microRNAs and their experimentally validated targets

Publically available datasets were examined for differential expression of microRNAs in human skeletal muscle during ageing (Drummond et al. 2011b; Rivas et al. 2014a) in order to establish whether a specific set of miRNAs was affected by ageing in both mouse and human skeletal muscle. Only 7 microRNAs were consistently deregulated during ageing in human and mouse skeletal muscle (Table 1; miRs highlighted in red). Ingenuity pathways analysis indicated that differential expression of these 7 microRNAs and consequently their target genes may result in defective insulin, MAPK and TGFβ signalling, with microRNA-181a being central to controlling these interactions. Interestingly, miR-181a is predicted to target several hundred genes (Figs. 2, S3), including genes associated with p38, NF-kB and TGFβ signalling, as well as genes previously reported to play an important role in skeletal muscle, such as *Sirt1,Pten* and *Nfatc1* (Figs. 2, S3).

**Fig. 3:**
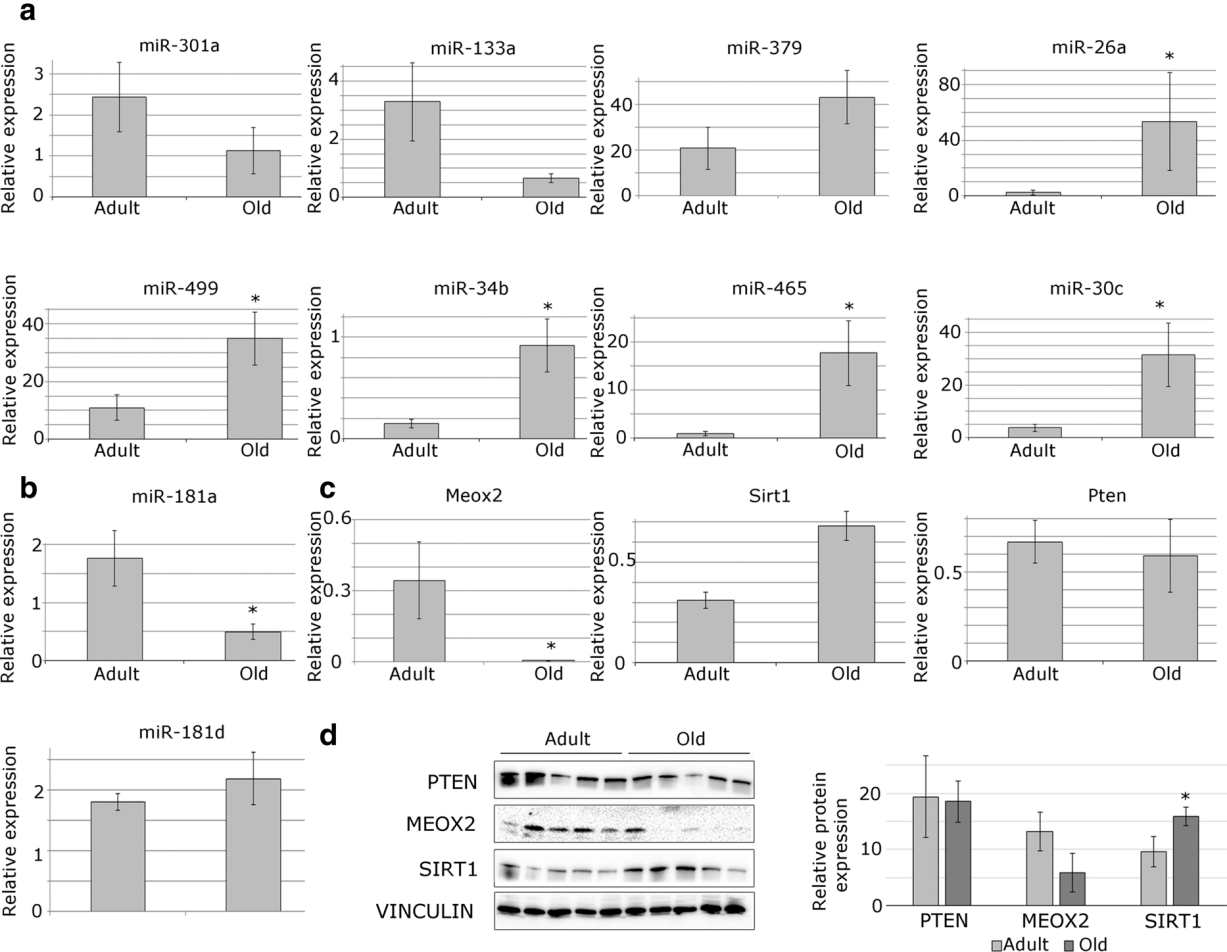
Validation of the differential expression of miRNAs and target mRNAs in muscle during ageing. **a** qPCR showing expression (relative to Rnu-6) of miRs differentially expressed in skeletal muscle of mice and/or humans. **b** qPCR showing relative expression of miR-181a and miR-181d relative to Rnu-6 in TA muscle of mice during ageing. **c** qPCR showing relative (to *β-2-microglobulin*) expression of miR-181a predicted target genes in muscle of mice during ageing. **d** Western blot and quantification showing differential protein expression of miR-181a predicted targets in TA muscle of mice during ageing. Representative Western blots are shown. *Error bars* show SEM; n = 4–7; *p < 0.05

qPCR was used to validate the differential expression of the 7 microRNAs in the skeletal muscle of mice during ageing. The differential expression of miR-181a, miR-133a, miR-26a, miR-499, miR-34b and miR-30c was validated in muscle during ageing (Fig. 3a, b). Since miR-181a, but not miR-181d, expression was significantly affected by ageing and it was predicted to have a central role in the microRNA:mRNA interactions affected by ageing, we analysed the expression of several miR-181a predicted target genes associated with muscle function by qPCR and western blot (Fig. 3c, d). We were unable to detect significant changes in the expression of *Pten* and *Meox2* at the mRNA and/or protein level (Fig. 3c, d). Among the analysed miR-181a predicted target genes, SIRT1 protein levels were significantly affected by ageing (Fig. 3d, e). miR-181a and SIRT1 protein expression were inversely correlated in muscle during ageing suggesting that *Sirt1* may be one of the key miR-181a targets in skeletal muscle during ageing.

### miR-181a directly regulates the expression of *Sirt1*

**Fig. 4:**
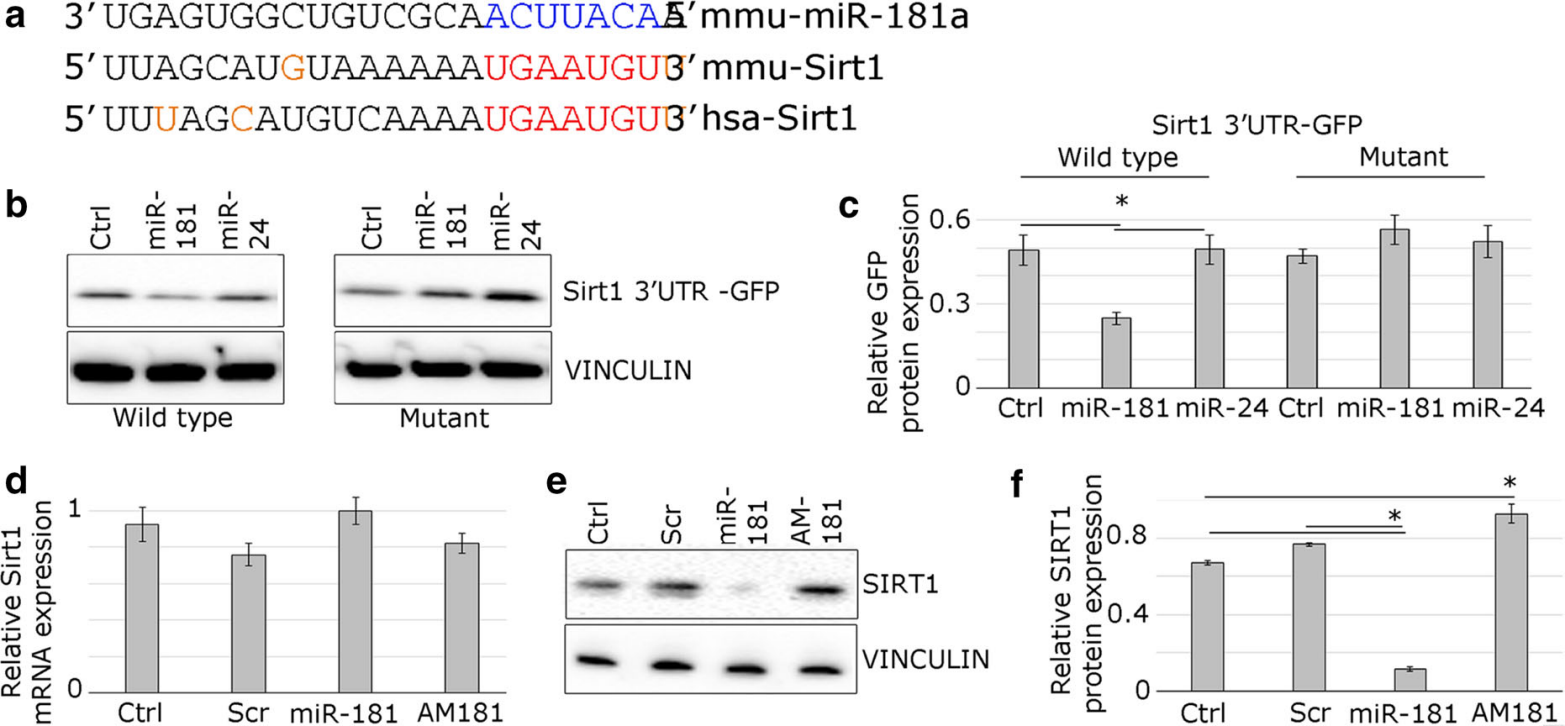
miR-181a represses *Sirt1* expression in myotubes. **a** Alignment of putative miR-181a target site in the 3′UTR of *Sirt1* gene; human and mouse sequence are indicated; conserved miR-181a putative target site is indicated in red; complementary nucleotides are shown in orange, miR-181a seed sequence is shown in blue. **b** GFP-*Sirt1* 3′UTR sensor constructs containing conserved mouse wild type or mutated miR-181a target site were transfected into mouse myoblasts. Co-transfection with miR-181a mimic but not miR-24 mimic, led to downregulation of GFP protein expression compared to mock-transfected control (Ctrl), as shown by representative western blot. Point mutations in the microRNA target site (mutant) rendered the sensor construct unresponsive. **c** Quantification of western blots is shown. **d**, **e** Endogenous SIRT1 protein but not mRNA expression is regulated by miR-181 in C2C12 myotubes, as shown by representative Western blot or qPCR, respectively. **f** Quantification of Western blots is shown. *Error bars* show SEM; *p < 0.05 (compared with control or scrambled control as indicated); n = 3

The 3′UTR of *Sirt1* has one putative miR-181 binding site conserved between human and mouse (Fig. 4a). To establish whether miR-181 directly interacts with the *Sirt1* 3′UTR, we generated a reporter construct containing a fragment of the *Sirt1* 3′UTR downstream of a GFP reporter (“wild type”). “Mutant” reporter contained a mutated miR-181 binding site. The GFP reporter containing wild type *Sirt1* 3′UTR was efficiently regulated by miR-181 but not by miR-24; a microRNA not predicted to target *Sirt1* (negative control) (Fig. 4b, c). Mutation of the putative target site in the 3′UTR rendered the reporter construct insensitive to miR-181, indicating that interaction with the target site is required for the response (Fig. 4b, c).

To validate *Sirt1* as a physiologically relevant miR-181 target gene in muscle, the expression of *Sirt1* transcript and protein was examined in C2C12 myotubes following miR-181 overexpression or inhibition using miRNA mimic or antimiR (AM181), respectively (Fig. 4c, d). The efficiency of the transfections was validated (Fig. S4). The expression of SIRT1 protein, but not mRNA in C2C12 myotubes was downregulated following overexpression of miR-181 and upregulated following inhibition of miR-181 function (Fig. 4d–f). These data show that miR-181a directly regulated SIRT1 expression at the protein level.

### Changes in miR-181a:Sirt1 affect myotube size

**Fig. 5:**
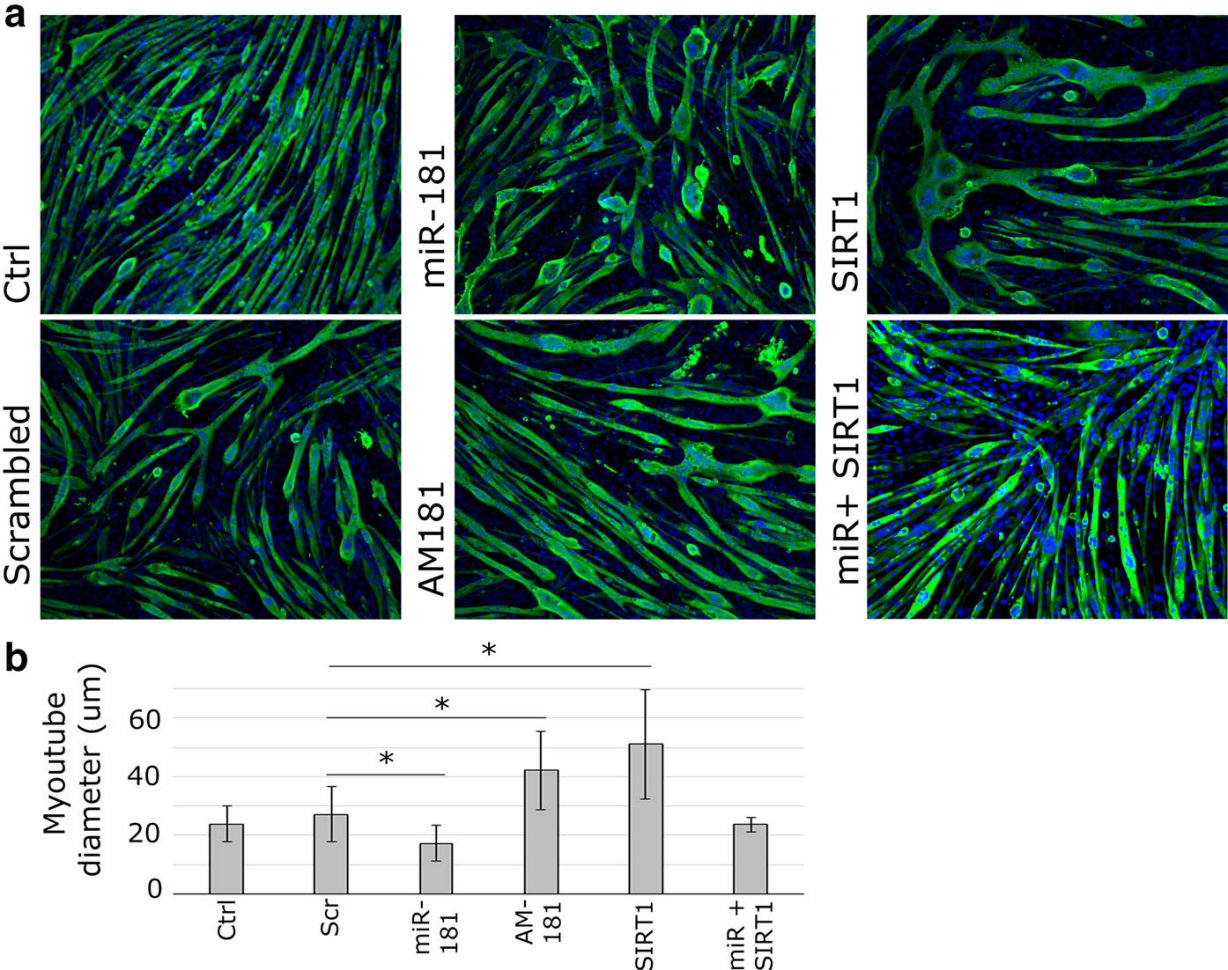
miR-181 negatively regulates myotube size by targeting *Sirt1*. Expression of miR-181 and its target gene, *Sirt1*, was manipulated in C2C12 myotubes; following transfections myotubes were stained for myosin heavy chain: MF20 - green; DAPI-blue. **a** MF20 immunostaining showing myotube size regulation by miR-181a and *Sirt1*. **b** Quantification of myotube diameter is shown (µm). Error bars show SEM, *p < 0.05 (compared to control), n = 4

To establish whether age-related changes in miR-181a expression may have functional consequences on muscle homeostasis, C2C12 myotubes were used as an in vitro model to study myotube hypertrophy and/or atrophy. C2C12 myotubes were transfected with miR-181 mimic or inhibitor (AM) or SIRT1 overexpression construct. At 4 days following transfection myotubes were stained for myosin heavy chain (MF20) and myotube diameter measured. miR-181a overexpression led to a significant decrease in myotube diameter, whereas miR-181a inhibition led to an increase in myotube diameter as compared to mock- and scrambled-transfected controls (Fig. 5). SIRT1 overexpression resulted in an increased myotube diameter as compared to mock- and scrambled- transfected controls (Fig. 5). Co-transfection of SIRT1 overexpression construct together with miR-181 mimic rescued the miR-181-induced phenotype, indicating the importance of *Sirt1* as miR-181 target gene in controlling myotube size (Fig. 5).

The effect of changes in miR-181 and SIRT1 expression on the expression of *p21*, a cell cycle regulator associated with senescence and upregulated in muscle of older mice, was examined (Table 2; Fig. S5). We did not detect significant changes in *p21* mRNA expression following manipulation of miR-181a levels, however *p21* expression was downregulated following SIRT1 upregulation in C2C12 myotubes indicating that SIRT1 may play additional, miR-181-independent function in muscle, such as regulating cell senescence (Fig. S5).

These data suggest that changes in miR-181a and expression of its target gene *Sirt1* in skeletal muscle during ageing may indeed have functional consequences on muscle homeostasis, specifically myofibre hypertrophy and/or atrophy.

## Discussion

The molecular mechanisms involved in sarcopenia development are not fully understood, however it is clear that transcriptomic, proteomic and epigenetic changes are involved. miRNAs can simultaneously modulate many signalling pathways and are therefore likely to be high-throughput regulators of pathophysiological changes associated with sarcopenia.

We have identified and validated miRNAs and mRNAs differentially expressed in skeletal muscle during ageing. In mouse muscle (TA), 30 miRNAs were differentially expressed during ageing (Table 1). Of these, 7 miRNAs were differentially expressed during ageing in the skeletal muscle of mice and humans based on data presented here and deposited in publically available databases (Drummond et al. 2011b; Rivas et al. 2014a). 5 microRNAs with a role in muscle biology: miR-26a (Dey et al. 2012), miR-499 (van Rooij et al. 2009a), miR34b (Roberts et al. 2012), miR-30c (Ketley et al. 2013) and miR-181a (Naguibneva et al. 2006) were validated as differentially expressed during ageing in the skeletal muscle of mice (Fig. 3a). The differential expression of the miRNAs during ageing was validated in whole muscle rather than myofibres and therefore it is possible that age-related changes in muscle quality, such as adipocyte or immune cell infiltration, may be associated with changes in microRNA expression. Nevertheless, these data suggest that deregulation of microRNA expression during ageing may have functional consequences related to disrupted muscle homeostasis.

Previous studies have investigated changes in the expression of miRNAs in muscle during ageing (Drummond et al. 2011b; Hu et al. 2014b; Kim et al. 2014b), however it is noteworthy, that the overlap in deregulated miRNA expression in muscle of old animals between different studies is limited. This may be due to the use of different transcriptomic platforms or differential ageing processes in anatomically distinct muscle types. Standardising research methods used to globally profile changes in miRNA expression in different types of muscle and organisms would be an important step forward towards a better understanding of the role of microRNAs in the decline of muscle mass and function during ageing.

Using miRSystem and Ingenuity pathways, we modelled microRNA:target interactions affected by ageing. Our data indicates that deregulated miRNA:target interactions may be responsible for ageing-related pathophysiological processes in skeletal muscle through regulation of ubiquitin-mediated proteolysis, ER stress and NFκB signalling, senescence and splicing factor expression, previously reported to be affected by ageing (Carvalho et al. 1996; Brack et al. 2007; Deldicque 2013; Holly et al. 2013; Drummond et al. 2014). Interestingly, *Ubc, Ucp3* and IGF signalling pathways were also predicted to be a part of the disrupted microRNA:target interactions during ageing (Fig. S2). As the anabolic action of insulin is reduced in muscle during ageing, and as muscle absorbs large amounts of circulating glucose, changes in IGF signalling in muscle during ageing could affect the levels of circulating glucose and therefore glucose present in other tissues, therefore affecting metabolism (Rasmussen et al. 2006).

Further analysis of the interactome of miRNAs differentially expressed during ageing in mouse and human muscle revealed that insulin, MAPK and TGFβ signalling, as well as ER stress, are likely to be regulated by these microRNAs; with miR-181a potentially playing a key role in age-associated changes in gene expression and signalling pathways in muscle. The analysis of miR-181a predicted targets supports this hypothesis, as miR-181a predicted target genes include genes/pathways with known role(s) in maintaining muscle homeostasis, for example *Sirt1*, *Pten*, and *p38*, *Tgfβ*, *Tnfα*, NF-kB and insulin signalling pathways (Fig. S3). miR-181a has been previously shown to regulate muscle regeneration (Naguibneva et al. 2006) and downregulation of its expression may be also related to deterioration of satellite cell function and impaired regeneration of muscle during ageing.

miRNAs provide a potent and highly responsive mechanism enabling cells to react to changes in their environment by controlling cellular protein content through regulating the expression of multiple target genes. It is therefore important to characterise miRNA target gene(s) when establishing miRNA function. We have validated differential expression of miR-181a and mRNA and analysed protein expression of miR-181a predicted targets genes: *Sirt1*, *Pten*, *Meox2*, in the mouse TA muscle during ageing (Fig. 3). *Sirt1* is a member of the sirtuin family, a NAD+ -dependent protein deacetylase known for its protective anti-ageing effects and also shown to promote muscle hypertrophy (Lee and Goldberg 2013; Sin et al. 2015). *Meox2* controls muscle size and myofibre metabolism (Otto et al. 2010), whilst *Pten* inhibition improves muscle regeneration (Hu et al. 2010). Among miR-181a predicted targets, SIRT1 protein levels were significantly changed in muscle during ageing (Fig. 3). We have validated *Sirt1* as a physiologically relevant direct miR-181a target in C2C12 myotubes (Fig. 4) and showed that miR-181 regulates myotube size through *Sirt1*, and potentially other target genes (Fig. 5). Since the expression of miR-181a is downregulated and *Sirt1* expression is upregulated in muscle during ageing, and miR-181 negatively regulates myotube size, we suggest that age-related changes in miR-181a and its target gene(s) expression may act as a failing compensatory mechanism intended to preventing loss of muscle mass and potentially function. Previous data has shown that such compensatory mechanisms exist. For example, elevated levels of miR-206 in muscle of the amyotrophic lateral sclerosis (ALS) mouse model have been shown to compensate for the decreased neuromuscular interactions (Williams et al. 2009). Future experiments using in vivo model organism(s) will determine whether age-related changes in miR-181a expression may indeed act as a compensatory mechanism to maintain muscle mass and potentially function during ageing. It is noteworthy that Hu et al. (2014a, b) have shown that deregulation of miR-29 expression in muscle during ageing is associated with increased muscle senescence and therefore it is likely that changes in microRNA expression during ageing might act as compensatory or causative events, depending on the specific microRNA.

miRNAs present a potential to regulate a variety of pathophysiological conditions, such as sarcopenia. The ability to manipulate miRNA expression may offer therapeutic potential for ameliorating sarcopenia by modulating the rate of loss of muscle mass and function with age. However, it is necessary to establish the nature of changes in miRNA expression during ageing, as well as the factors triggering these changes. Furthermore, functional studies are needed to establish whether miRNAs initiate ageing-related changes in muscle homeostasis or fine-tune an already initiated process, and whether some microRNAs act in a compensatory manner to maintain muscle homeostasis in older individuals.

## Electronic supplementary material

Below is the link to the electronic supplementary material.

Fig. S1 The relative log-expression (RLE) plots shown for **a** gene expression and (**c**) microRNA expression demonstrate that the data distributions were consistent across all of the arrays, without the presence of a clear outlier or technical effect on variability. Both RLE plots indicate that these datasets satisfy the assumptions of the robust multi-chip averaging (RMA) algorithm used for further processing of the arrays. **b**, **d** The volcano plots show the statistical significance of differential expression (log_10_ p values) against the extent of differential expression (log_2_ fold-change), for both gene- **b** and microRNA -**d** expression datasets and demonstrate that there are a large number of interesting genes/microRNAs (shown in *blue*) that are significantly differentially expressed between adult and old mice, according to the threshold levels used for p value and log_2_ fold-change (p < 0.05, log_2_ fold-change > 0.5). [*Statistical significance was assessed using an empirical Bayes moderated t-test*]. Plots were generated in R using functions from ggplot2 and oligo. Supplementary material 1 (JPEG 2011 kb)

Fig. S2 Disrupted microRNA:target interaction networks in skeletal muscle of mice during ageing. The network of interactions was constructed using IPA software. The input was microRNAs and their experimentally validated targets and mRNAs differentially expressed in muscle during ageing. In red miRs/genes that were downregulated with age are shown; in green miRs/genes that were upregulated with age are shown. Supplementary material 2 (JPEG 3354 kb)

Fig. S3 Predicted miR-181a targets are shown. The network of interactions was constructed using IPA software. The input was miR-181a predicted targets (with high confidence). Supplementary material 3 (TIFF 16208 kb)

Fig. S4 **a** miR-181 expression can be modulated in C2C12 myotubes. qPCR showing miR-181a expression relative to Rnu-6 following mock transfection or transfections with scrambled antimiR, miR-181a mimic or antimiR-181a. **b** Ct values for β-2-microglobulin qPCR showing that the expression of this gene is stable in the TA muscle of mice during ageing. Supplementary material 4 (TIFF 2558 kb)

Fig. S5 miR-181 does not control the expression of p21, a marker of senescence. **a** p21 expression is upregulated in the muscle of old mice compared to muscle of adult mice as shown by qPCR. **b** SIRT1, but not miR-181a upregulation or inhibition had an effect on p21 mRNA expression in C2C12 myotubes as shown by qPCR. Expression relative to β-2-microglobulin is shown. *Error bars* show SEM; *p < 0.05; n=4–6. Supplementary material 5 (JPEG 140 kb)

Table S1 Sequences of primers used.
Table S2 List of reagents used. Supplementary material 6 (DOCX 13 kb)
