# Supplementary figures and images for: The functional consequences of age-related changes in microRNA expression in skeletal muscle
Source: Biogerontology. 2016 Feb 27;17:641–54. doi: 10.1007/s10522-016-9638-8 (PMC4889642; doi:10.1007/s10522-016-9638-8)

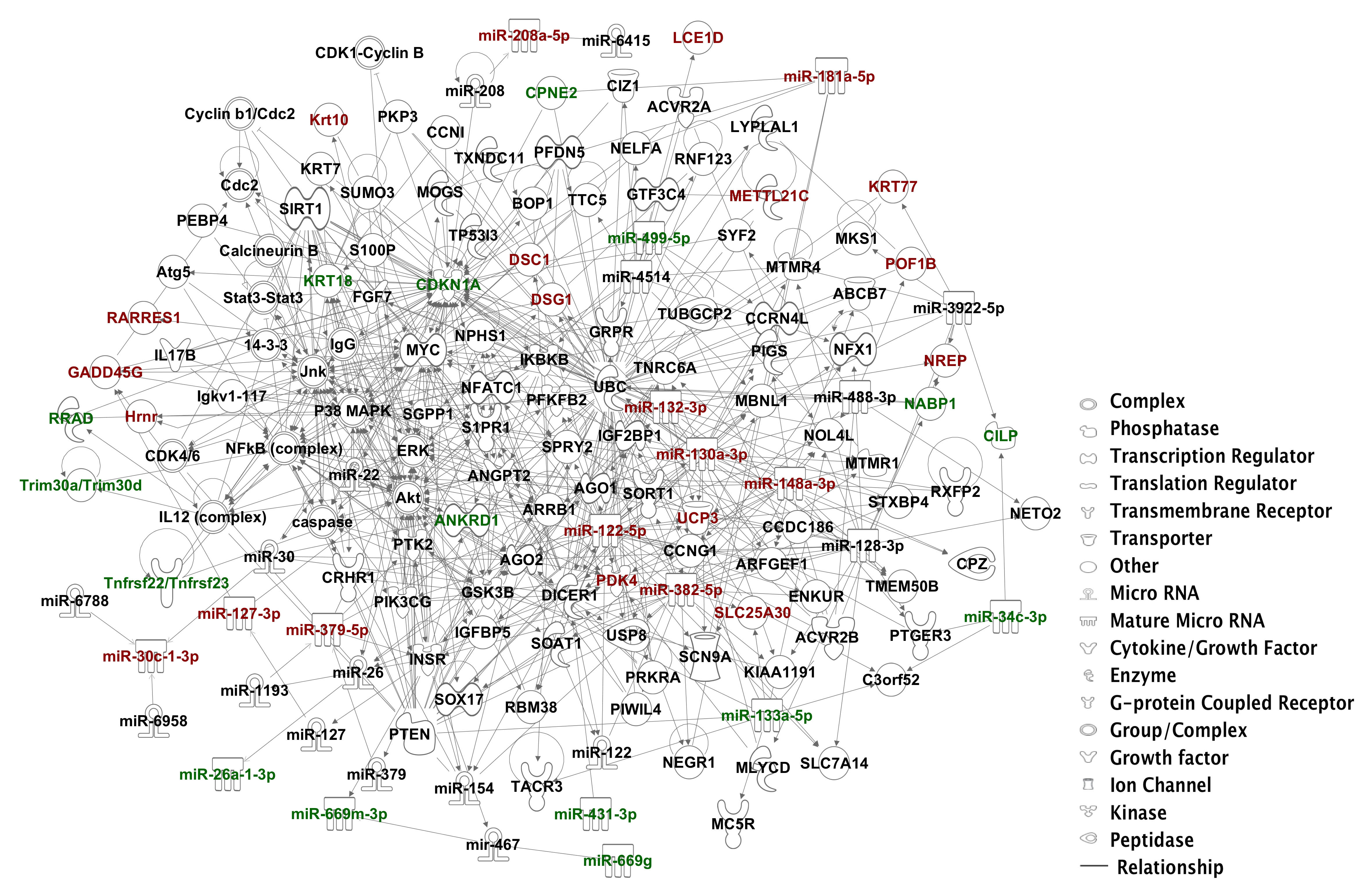

Supplement: Supplementary file 1 — Fig. S1 The relative log-expression (RLE) plots shown for a gene expression and (c) microRNA expression demonstrate that the data distributions were consistent across all of the arrays, without the presence of a clear outlier or technical effect on variability. Both RLE plots indicate that these datasets satisfy the assumptions of the robust multi-chip averaging (RMA) algorithm used for further processing of the arrays. b, d The volcano plots show the statistical significance of differential expression (log10 p values) against the extent of differential expression (log2 fold-change), for both gene- b and microRNA -d expression datasets and demonstrate that there are a large number of interesting genes/microRNAs (shown in blue) that are significantly differentially expressed between adult and old mice, according to the threshold levels used for p value and log2 fold-change (p < 0.05, log2 fold-change > 0.5). [Statistical significance was assessed using an empirical Bayes moderated t-test]. Plots were generated in R using functions from ggplot2 and oligo. Supplementary material 1 (JPEG 2011 kb) [file 10522_2016_9638_MOESM1_ESM.jpg]

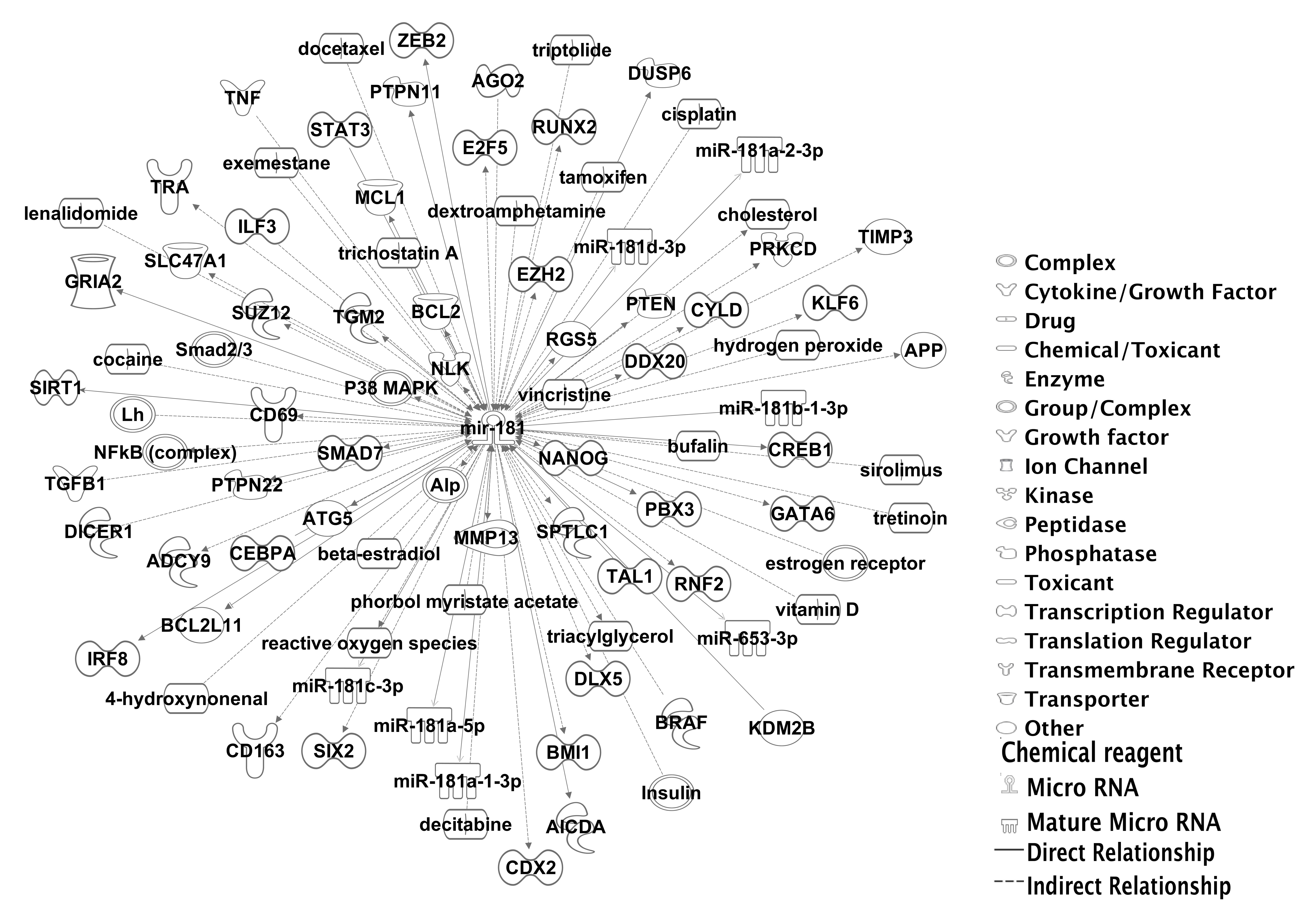

Supplement: Supplementary file 2 — Fig. S2 Disrupted microRNA:target interaction networks in skeletal muscle of mice during ageing. The network of interactions was constructed using IPA software. The input was microRNAs and their experimentally validated targets and mRNAs differentially expressed in muscle during ageing. In red miRs/genes that were downregulated with age are shown; in green miRs/genes that were upregulated with age are shown. Supplementary material 2 (JPEG 3354 kb) [file 10522_2016_9638_MOESM2_ESM.jpg]

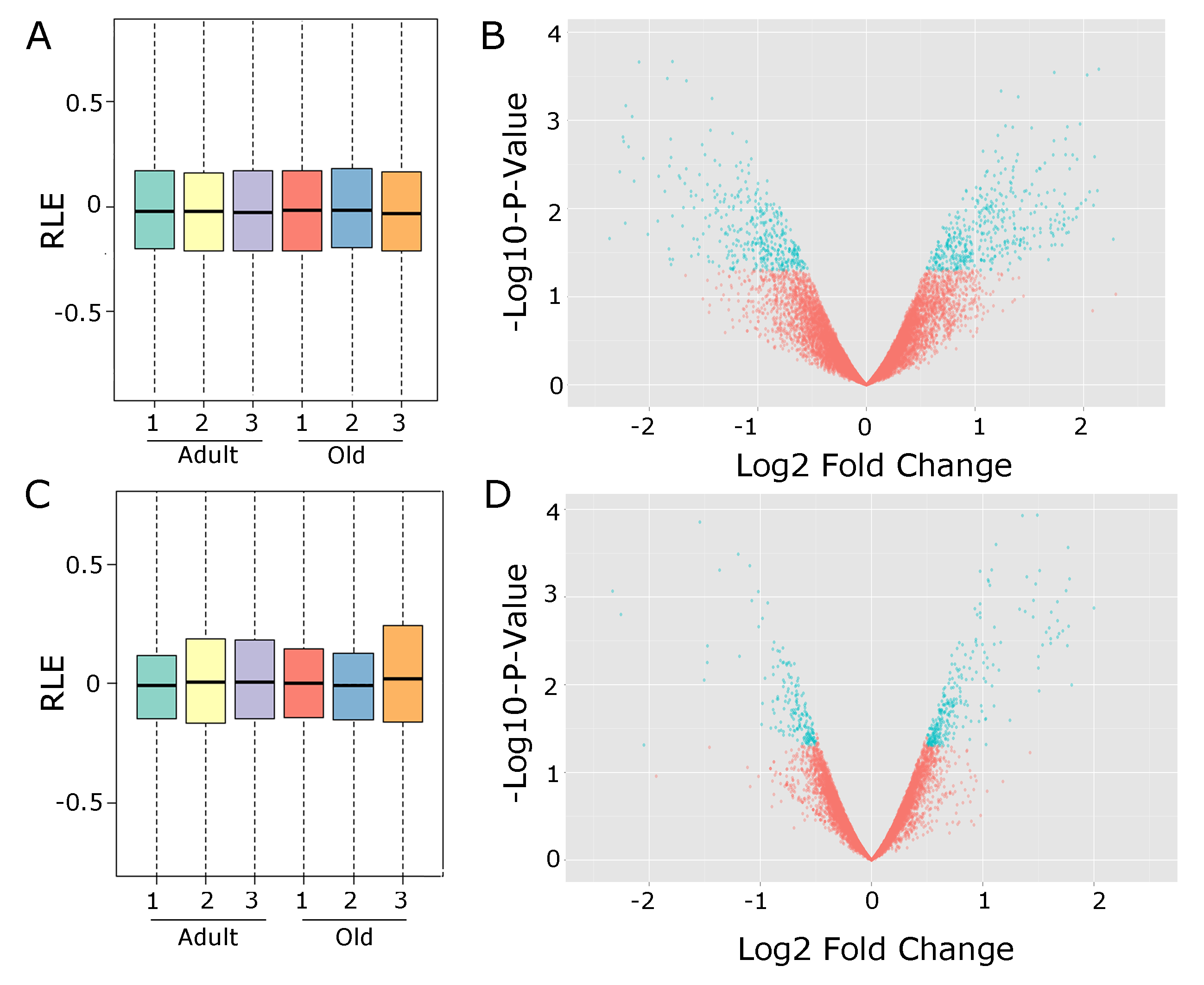

Supplement: Supplementary file 3 — Fig. S3 Predicted miR-181a targets are shown. The network of interactions was constructed using IPA software. The input was miR-181a predicted targets (with high confidence). Supplementary material 3 (TIFF 16208 kb) [file 10522_2016_9638_MOESM3_ESM.tif]

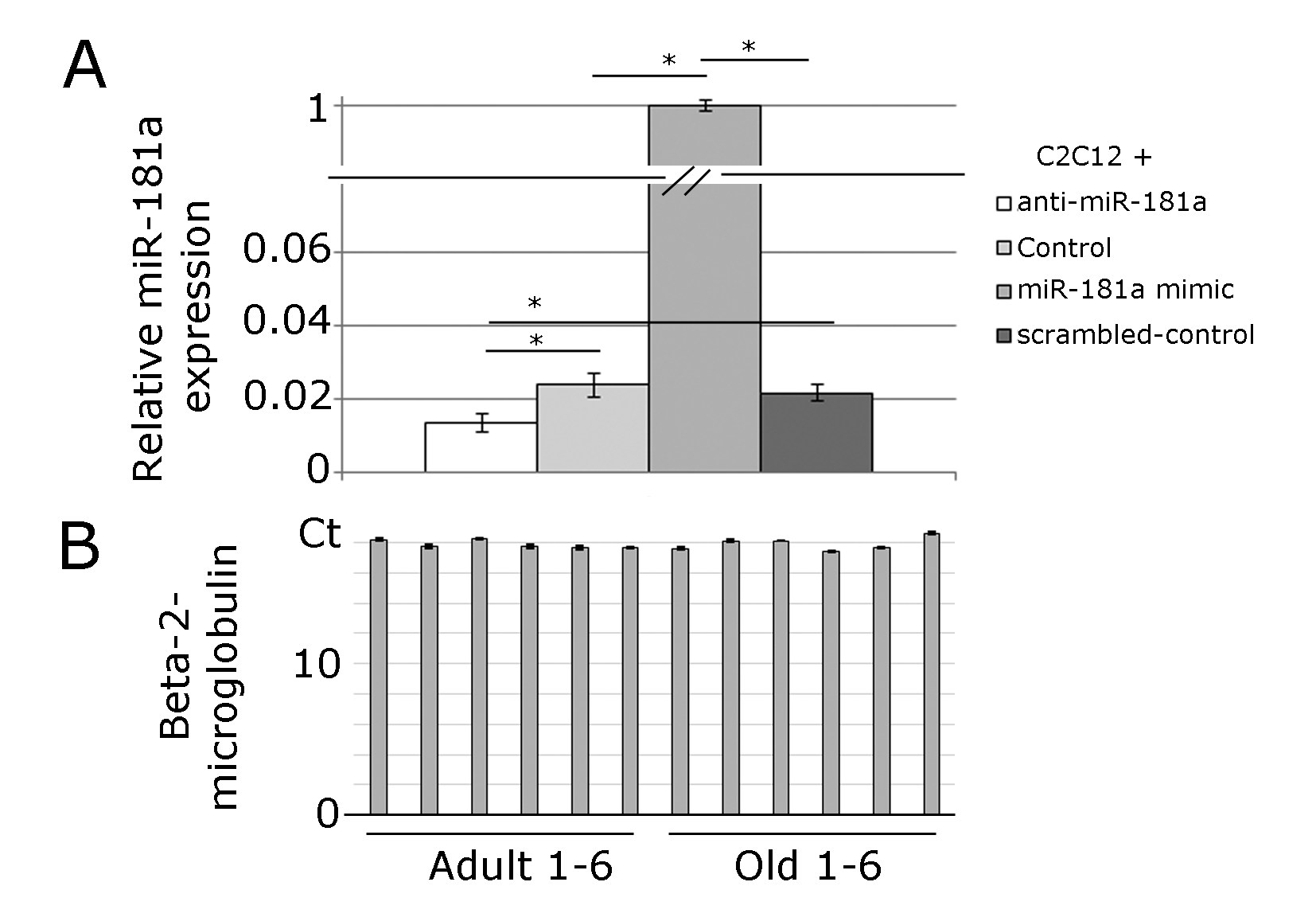

Supplement: Supplementary file 4 — Fig. S4 a miR-181 expression can be modulated in C2C12 myotubes. qPCR showing miR-181a expression relative to Rnu-6 following mock transfection or transfections with scrambled antimiR, miR-181a mimic or antimiR-181a. b Ct values for β-2-microglobulin qPCR showing that the expression of this gene is stable in the TA muscle of mice during ageing. Supplementary material 4 (TIFF 2558 kb) [file 10522_2016_9638_MOESM4_ESM.tif]

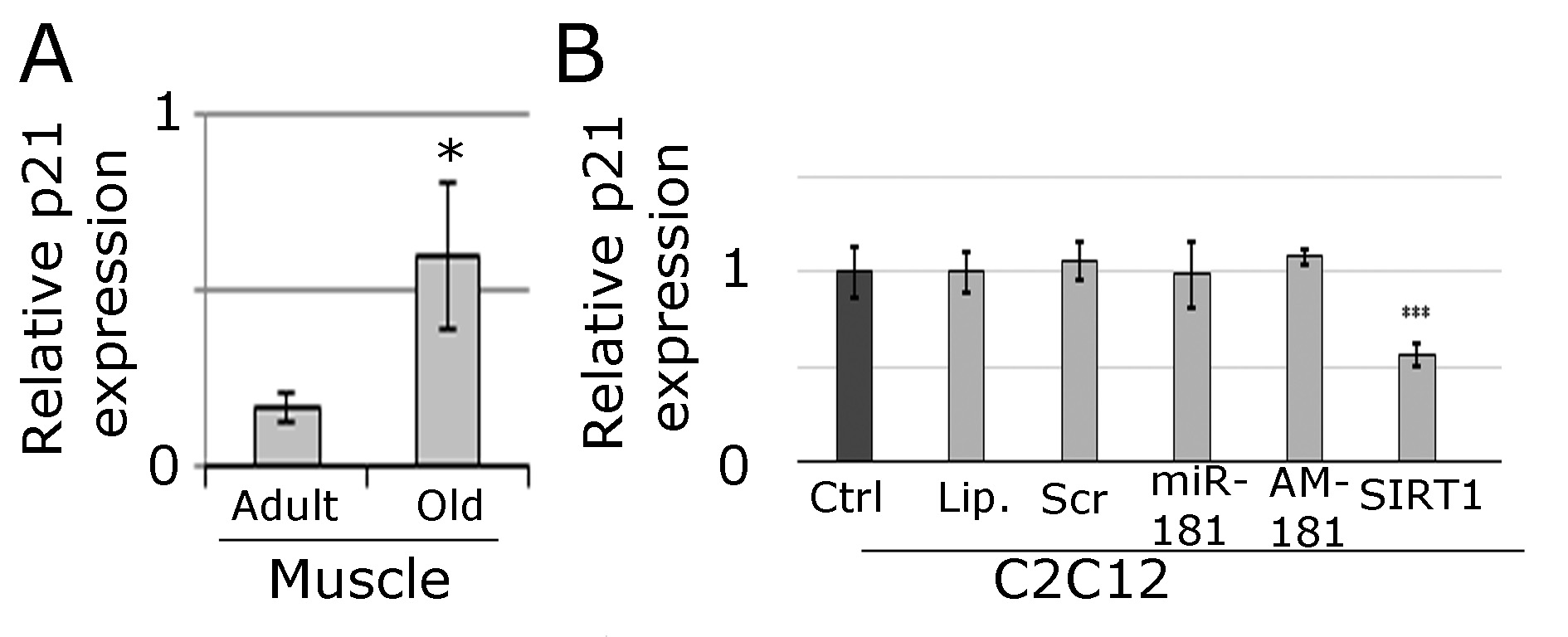

Supplement: Supplementary file 5 — Fig. S5 miR-181 does not control the expression of p21, a marker of senescence. a p21 expression is upregulated in the muscle of old mice compared to muscle of adult mice as shown by qPCR. b SIRT1, but not miR-181a upregulation or inhibition had an effect on p21 mRNA expression in C2C12 myotubes as shown by qPCR. Expression relative to β-2-microglobulin is shown. Error bars show SEM; *p < 0.05; n=4–6. Supplementary material 5 (JPEG 140 kb) [file 10522_2016_9638_MOESM5_ESM.jpg]
