# Supplementary material for: The functional consequences of age-related changes in microRNA expression in skeletal muscle
Source: Biogerontology. 2016 Feb 27;17:641–54. doi: 10.1007/s10522-016-9638-8 (PMC4889642; doi:10.1007/s10522-016-9638-8)
Supplement: Supplementary file 6 — Table S1 Sequences of primers used. Table S2 List of reagents used. Supplementary material 6 (DOCX 13 kb) [file 10522_2016_9638_MOESM6_ESM.docx]

| **Gene** | **Forward primer** | **Reverse primer** |
| --- | --- | --- |
| Sirt 1 | TGACCGATGGACTCCTCACT | ACAAAAGTATATGGACCTATCCGC |
| Meox2 | GGAAAAGCGACAGTTCAGATTCCCAA | GCCCCTTGCTGGCCTCCTTT |
| Pten | GCTTCTGCCATCTCTCTCCTC | AATGTCTCTCAGCACATAGATTGT |
| Beta-2-microglobulin | GGAGAATGGGAAGCCGAACA | TCTCGATCCCAGTAGACGGT |
| P21 | ATCCAGACATTCAGAGCCACAG | TCGGACATCACCAGGATTGG |
| Nfatc1 | CGGCCGCAGAACACTACA | TGGCTGAAGGAACAGCTGAG |
| Hdac9 | AGCAATAAGGAAAAGGCTGGGA | TCAGAAGGGCTGACGGTTG |

**Table S1.**

| **Product** | **Product number** | **Source** |
| --- | --- | --- |
| **Antibodies** |  |  |
| MF20 antibody | MF20 | Developmental Studies Hybridoma Bank |
| SIRT1 antibody | Ab12193 | Abcam |
| VINCULIN antibody | Ab73412 | Abcam |
| Meox2 antibody | Ab117551 | Abcam |
| GFP antibody | 10362 | Life Technologies |
| PTEN antibody | Ab32199 | Abcam |
| **Transfections, cell culture** |  |  |
| miR-181a mimic | MSY0000210 | Qiagen |
| miR-181a inhibitor (antimiR) | MIN0000210 | Qiagen |
| mR-24 mimic | MSY0000219 | Qiagen |
| Lipofectamine2000 | 11668027 | Life Technologies |
| SA-b-Galactosidase staining kit | 9860 | Cell Signalling Technology |
| GFP TOPO | K4810-01 | Life Technologies |
| **Real-Time PCR** |  |  |
| Superscript II | 18064 | Life Technologies |
| miRScript RT II | 218161 | Qiagen |
| miRScript SybrGreen | 218073 | Qiagen |
| Sso-Advanced SybrGreen | 172-5270 | Biorad |
| miR-181a qPCR primer | MS00011263 | Qiagen |
| RNU-6 qPCR primer | MS00033740 | Qiagen |
| Snord-61 qPCR primer | MS00033705 | Qiagen |
| miR-133a primer | MS00032305 | Qiagen |
| miR-301a primer | MS00011683 | Qiagen |
| miR-26a primer | MS00032613 | Qiagen |
| miR-499 primer | MS00002576 | Qiagen |
| miR-34b primer | MS00011900 | Qiagen |
| miR-465 primer | MS00012082 | Qiagen |
| miR-379 primer | MS00011942 | Qiagen |
| miR-30c primer | MS00011725 | Qiagen |
| miR-181d |  |  |

**Table S2.**
